# A New Mode of Setting Artificial Teeth

**Published:** 1852-01

**Authors:** A. Hill


### Selections.

From the N. Y. Dental Recorder.

### A NEW MODE OF SETTING ARTIFICIAL TEETH.

The undersigned has invented and put in operation a plan by which artificial teeth may be mounted, and secured in the mouth without the aid of metallic plates or springs of any kind. This devise consists of an artificial compound substance, (similar in its composition to "*Hill's Stopping*,") that can easily be moulded while in a plastic state, over a plaster model of the mouth, and of a color resembling the natural gums. This substance can be moulded with perfect facility to any desired form, and soon becomes sufficiently firm and solid to hold the teeth in their places and sustain them in the mouth. Being formed in its plastic state, it can be made to fit any model in the most perfect manner, so that *even single* teeth, or *partial* sets, are easily retained in the mouth without the aid of ordinary springs or clasps, by means of atmospheric pressure alone.

The great ease and facility of working this substance, enables the operator to extend it in any direction, and to mould it over the alveolar ridge in such a manner as to restore the contour of the face, no matter to how great an extent absorption of the ridge may have taken place. It likewise obviates the necessity of gum-teeth altogether, as a beautiful artificial gum may be easily formed of the substance itself.

The teeth are secured by moulding the compound around their base, without the necessity of backing or soldering in the ordinary way, and are easily restored in case of accident, without interfering with the rest.

Entire or partial sets can be made with equal facility, and worn with great ease and comfort, as a *perfect* fit is readily obtained, and no sharp cutting edges are felt to irritate and fret the parts.

For *under* sets, it cannot fail to commend itself, where firmness and solidity are so desirable, because of its extreme simplicity, and convenience. For here the edges of the piece that rest upon the muscles, can be made just as obtuse or round as the operator may desire, and the circumstances of the case admit, and the alveolar ride built up to any desirable form.

In short, this arrangement seems to us, to reduce the difficult process of mounting artificial teeth, to the greatest possible degree of simplicity, and we are at a loss to conceive how any mode, *more simple or economical,* can possibly be devised.

We are now ready to anticipate some of the questions and objections that will doubtless be started in the minds of our readers.

1st. Is the compound firm enough to sustain the teeth in mastication?

We answer yes, abundantly so in most cases, and *especially* so where circumstances will allow a considerable degree of thickness over the alveolar border, and *almost every case* will allow this.

2d. Will it withstand the fluids of the mouth?

We answer—It will stand as well as "Hill's Stopping," and *that* will stand where there is no friction from mastication.

But, "the proof of the pudding, (as the old adage says,) is in eating it," and *time alone* can test the durability of this arrangement. We have several pieces that are now being worn by our patients very successfully. One piece has been worn, without the *least alteration*, for more than six months, and that, too, under circumstances calculated to test it *severely*, and is likely to last an indefinite length of time to come.

For *temporary* sets, we are *certain* they must succeed, and we have high hopes of something more, but time alone must settle this question.

For several months past we have been experimenting with respect to a *gum color*, and have succeeded so well that we expect soon to offer it to the profession, on such terms as may seem to us *just* and proper. A. Hill.

*October 22d,* 1851.

